# Autophagy-Related Gene 4 Participates in the Asexual Development, Stress Response and Virulence of Filamentous Insect Pathogenic Fungus *Beauveria bassiana*

**DOI:** 10.3390/jof9050543

**Published:** 2023-05-06

**Authors:** Jin-Li Ding, Kang Wei, Ming-Guang Feng, Sheng-Hua Ying

**Affiliations:** Institute of Microbiology, College of Life Sciences, Zhejiang University, Hangzhou 310058, China; 22207009@zju.edu.cn (K.W.);

**Keywords:** autophagy-related gene 4, conidiation, dimorphism, stress tolerance, virulence, *Beauveria bassiana*

## Abstract

Autophagy is a conserved mechanism for the turnover of intracellular components. Among the ‘core’ autophagy-related genes (*ATGs*), the cysteine protease Atg4 plays an important role in the activation of Atg8 by exposing the glycine residue at its extreme carboxyl terminus. In the insect fungal pathogen *Beauveria bassiana*, a yeast ortholog of Atg4 was identified and functionally analyzed. Ablation of the *BbATG4* gene blocks the autophagic process during fungal growth under aerial and submerged conditions. Gene loss did not affect fungal radial growth on various nutrients, but Δ*Bbatg4* exhibited an impaired ability to accumulate biomass. The mutant displayed increased sensitivity to stress caused by menadione and hydrogen peroxide. Δ*Bbatg4* generated abnormal conidiophores with reduced production of conidia. Additionally, fungal dimorphism was significantly attenuated in gene disruption mutants. Disruption of *BbATG4* resulted in significantly weakened virulence in topical and intrahemocoel injection assays. Our study indicates that BbAtg4 contributes to the lifecycle of *B. bassiana* via its autophagic roles.

## 1. Introduction

*Beauveria bassiana* is a natural enemy of various arthropod species by causing white muscardine disease and has been considered a potential alternative to chemical insecticides in pest management programs [1,2]. In the natural environment, *B. bassiana* produces conidia as infectious cells. Conidia germinate on the host cuticle via mobilization of endogenous reserves and develop into invasive hyphae [3,4]. The invasive hyphae penetrate through the host cuticle and proliferate in the host hemocoel via dimorphic change, generating yeast-like hyphal bodies (in vivo blastospore) [5,6]. After killing the hosts, *B. bassiana* efficiently utilizes the insect cadaver to support the saprotrophic growth and conidiation [7]. In eukaryotes, autophagy is an essential mechanism to regulate cellular homeostasis through degrading superfluous or damaged macromolecules and organelles [8]. This cellular degradation pathway is involved in the entire lifecycle of *B. bassiana* [9].

The autophagic process involves a set of autophagy-related genes (ATGs), in which the ‘core’ ATG genes are indispensable for all autophagy-related processes and conserved in eukaryotes [10]. Atg1 (a serine/threonine protein kinase) forms an induction complex that initiates nucleation and phagophore formation. Autophagosome formation is complicated and involves Atg8-phosphatidylethanolamine (PE) conjugate as a major structural component. The formation of Atg8-PE is dependent on the ubiquitin-like conjugation system (ULCS), in which the cysteine protease Atg4 exposes the glycine residue at the extreme C terminus [11]. The biological functions of Atg4 homologs have been increasingly characterized in filamentous fungi. In *Aspergillus oryzae*, Atg4 is indispensable for autophagosome formation and is involved in the development of aerial hyphae into conidia [12]. In *Botrytis cinerea* (a plant pathogenic fungus), gene disruption of *ATG4* significantly compromises mycelial growth, conidiation, and virulence [13]. *Fusarium graminearum* is the causal agent of Fusarium head blight, and its Atg4 contributes to fungal development, deoxynivalenol production, and virulence [14]. In rice blast fungus *Magnaporthe oryzae*, the ATG4 gene contributes to appressorial maturation and, ultimately, to fungal pathogenesis [15]. In *Metarhizium robertsii* (an insect pathogenic fungus), deletion of *ATG4* does not block appressorial formation but significantly impairs fungal lipid accumulation and virulence [16]. These investigations indicate that Atg4 homologs exhibit divergent roles in fungal physiology. However, the roles of Atg4 remain unknown in *B. bassiana*.

In the present study, we identified and characterized a cysteine protease Atg4 in *B. bassiana* and determined its roles in the fungal lifecycle. The results demonstrated that the *BbATG4* loss resulted in attenuated phenotypes in autophagic process, development, stress response and virulence.

## 2. Materials and Methods

### 2.1. Strains, Media and Growth Conditions

The wild type of *B. bassiana* ARSEF2860 (Bb2860) was obtained from the U.S. Department of Agriculture Entomopathogenic Fungus Collection (Ithaca, NY, USA) [17]. The wild type (WT) and its derivative strains were maintained on SDAY (4% glucose, 1% peptone, and 1.5% agar plus 1% yeast extract) at 25 °C. *Escherichia coli* DH5α (Invitrogen, Waltham, MA, USA) was cultured in a Luria–Bertani medium with necessary antibiotics for plasmid construction. *Agrobacterium tumefaciens* AGL-1 for fungal transformation was cultured in YEB broth (*w*/*v*: 0.5% sucrose, 1% peptone, 0.1% yeast extract, and 0.05% MgSO_4_). Czapek-Dox agar (CzA) (3% glucose, 0.3% NaNO_3_, 0.1% K_2_HPO_4_, 0.05% KCl, 0.05% MgSO_4_, and 0.001% FeSO_4_ plus 1.5% agar) was used as the chemically defined medium in following experiments.

### 2.2. Bioinformatic Analysis of BbAtg4

Basic Local Alignment Search Tool (BLAST) (http://blast.ncbi.nlm.nih.gov/blast.cgi (accessed on 1 March 2023)) was used to identify BbAtg4 protein through the NCBI databases using *S. cerevisiae* Atg4 (P53867) as a query. The Atg4 orthologs were downloaded from NCBI databases, and their domain architectures were analyzed through the online portal SMART (http://smart.embl-heidelberg.de (accessed on 1 March 2023)). The Atg4 homologs in yeasts and filamentous fungi were clustered using the maximum likelihood method through the online program MEGA7 (http://www.megasoftware.net/ (accessed on 1 March 2023)).

### 2.3. Targeted Gene Disruption and Complementation

A disruption mutant of *BbATG4* was generated using a method of homologous replacement coupled with a fluorescence reporter [18]. All primers are included in Table S1. The primer pairs P1/P2 and P3/P4 were used to amplify 5′- and 3′-fragments of *BbATG4*, respectively. The resulting fragments were cloned into the restriction enzyme sites (*Xma*I/*Bam*HI and *Xba*I/*Hpa*I) in p0380-bar using the ClonExpress II One Step Cloning Kit (Vazyme Biotech, Nanjing, China), generating gene disruption vector (p0380-bar-BbAtg4). The full-length gene of *BbATG4* was amplified with the primer pair P5/P6 and inserted into the plasmid pPK2-NTC-GFP [19], generating the complementation vector (pPK2-BbAtg4-NTC-GFP). The resulting vector was transformed into fungal strains with the *Agrobacterium*-based transformation method. Putative gene disruption and complementation strains were screened by phosphinothricin (200 μg/mL) and nourseothricin (50 μg/mL), respectively, and identified via PCR analyses withprimer pair P7/P8. 

### 2.4. Visualizing Autophagic Flux in Fungal Strains

Fusion protein GFP-Atg8 (GA8) was used as a marker to track the autophagic process [9]. Plasmid p0380-GA8-sur was integrated into the wild type and eight gene disruption mutant strains. To visualize autophagy in the aerial mycelia, conidia of the indicated strain were inoculated on SDAY plates and cultured at 25 °C. The aerial mycelia were sampled at 3.5 d post-incubation. To obtain the submerged mycelia, the conidia were inoculated into SDB (SDAY without agar) and cultured for 2 d at 25 °C. The mycelial samples were stained with CMAC and examined under a fluorescent microscope.

### 2.5. Phenotypic Assays

Effects of the gene loss on fungal phenotypes, including conidial germination, vegetative growth, stress response and development, were evaluated among the wild-type, gene disruption and complemented mutant strains as described previously [20,21]. All experiments were repeated three times.

Conidial germination: Conidial germination was examined on GA (sucrose-peptone agar) and WA (water-ager) plates. The conidial suspension (100 µL, 5 × 10^7^ conidia/mL) was inoculated on the indicated plates. The germination levels on these two media were measured at 10 h and 24 h post-incubation, respectively. The morphology of fungal cells was recorded using microscopy.

Vegetative growth: Mycelial growth was assayed on the CzA plates modified with various carbon and nitrogen sources. Carbon sources (final concentration, *w*/*v*) included glucose (3%), sucrose (3%), fructose (3%), trehalose (3%), olive oil (0.5%) and oleic acid (0.2%). Nitrogen sources (final concentration, *w*/*v*) included NH_4_NO_3_ (0.5%) and urea (0.5%). The radial growth rate was tested by dripping conidial suspension (1 µL, 10^6^ conidia/mL) on the plate, and colony diameter was examined at 7 d post-incubation at 25 °C. To determine biomass, conidial suspension (100 µL, 1 × 10^6^ conidia/mL) was smeared on the cellophane attached tothe indicated plate. After the 7d incubation at 25 °C, biomass was determined after drying.

Stress responses: Fungal responses to oxidative stress were determined on a CzA plate supplemented with 0.02 mM menadione and 2 mM H_2_O_2_. A droplet (1 µL) of conidial suspension (10^6^ conidia/mL) was placed on the plate and incubated at 25 °C. The colony diameter was measured at 7 d post-incubation. CzA plates without stress chemicals were used as control.

Fungal development: Conidial production was determined on SDAY plates. Aliquots (100 μL of 10^7^ conidia/mL) were inoculated on SDAY plates and cultured for 7 d at 25 °C. Mycelial discs (5 mm in diameter) were suspended in 0.02% Tween-80 solution. Conidial concentration in suspension was quantified and used to calculate conidial yield (conidial number per square centimeter). In addition, the mycelia of the wild-type and autophagy-null mutants were sampled at 4 and 5 d post-incubation, respectively. The conidium-producing structures were examined under a microscope. Fungal development under submerged conditions was assayed in SDB medium (SDAY without agar). Conidia were inoculated into SDB at the final concentration of 10^5^ conidia/mL and incubated for 3 d at 25 °C on a shaker. The concentration of blastospores in broth was determined, and blastospore yield was shown as the spore number per ml of culture.

### 2.6. Insect Bioassay with Two Methods in Preparing Conidial Suspensioi5n

To examine fungal virulence, the *Galleria mellonella* larvae were used as the bioassay hosts, and each treatment included 30–35 larvae [22]. Fungal strains were cultured on SDAY plates for 7 d at 25 °C, and the resultant conidia were used as infectious inocula. Two methods were used in preparing conidial suspension. In method 1, mycelia and conidia were harvested from the plate and suspended in 0.02% Tween 80 solution, followed by violent votexing. The resultant mixture was filtered through the cotton column, and the filtrate was used to infect the hosts. In method 2, the resultant filtrate from method 1 was filtered through the microporous membrane (40 µm in pore size) [23]. The resultant suspension of two methods was used in two kinds of bioassay.I n the cuticle inoculation assay, insects were immersed in conidial suspension (10^7^ conidia/mL) for 10 s. In the intrahemocoel injection assay, conidial suspension (5 µL, 10^5^ conidia/mL) was injected into the host hemocoel. Tween-80 solution (0.02%) was used as a control. The daily-recorded mortality was used to calculate the median lethal time (LT_50_) by Kaplan–Meier method with a log-rank test for determining the difference between the paired survival trends.

In previous bioassays for autophagy-null mutants, the conidial suspension was prepared with method 1. To increase the comparability of bioassay results, we re-examined all autophagy-null mutants published in the past decade [4,9,24,25,26] with conidial suspension prepared with method 2. 

### 2.7. Statistical Analyses

All other phenotypic measurements for the wild-type, gene disruption and complementation strains were subjected to Student’s *t*-test, and the significance was determined if *p* < 0.05. Statistical analyses were performed with the software of GraphPad Prism 8 (GraphPad Software, Boston, MA, USA).

## 3. Results

### 3.1. Characterization and Molecular Manipulation of BbAtg4

**Figure 1:**
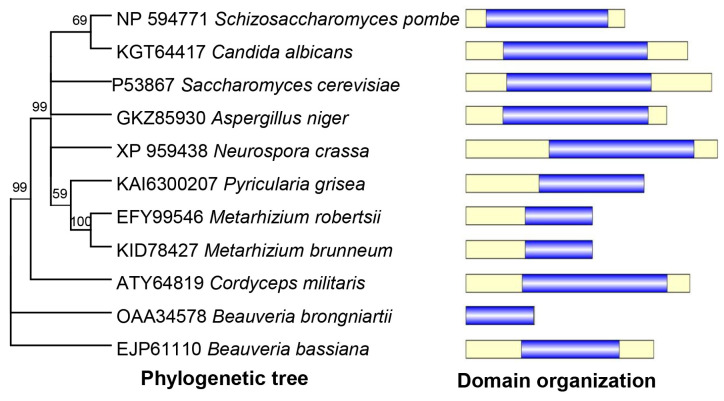
Sequence analyses for Atg4 protein in *B. bassiana*. Sequence analyses for Atg4 protein in *B. bassiana*. Phylogenetic relationship of *B. bassiana* Atg4 with its homologs in fungi. Relationships among different homologs were constructed by Neighbor-Joining analysis, and the numbers at each node indicated the bootstrap values > 50% from 1000 replicate tests. Each gene is indicated with GenBank accession number followed by the respective fungal species. Domain organization was shown for each homolog.

Based on the BLAST research with yeast Atg4 (Accession no. P53867) as a query, a highly related homolog (Accession no. EJP61110) was identified in *B. bassiana* and was designated as BbAtg4. The open reading frame (ORF) sequence of this gene was 1508 bp long, with three introns in the genomic sequence, and it coded a protein with 378 amino acids. Domain annotation analyses indicated that BbAtg4 contained a domain of Peptidase_C54 (PF03416). As shown in Figure 1, BbAtg4 was much more closely related to those of the filamentous fungi than to those of yeasts and showed more similarity to those of entomopathogenic fungi.

To further unveil the role of BbAtg4, the gene disruption strain was successfully constructed through the homologous recombination strategy (Supporting Information Figure S1A). The candidate transformants were screened by a PCR reaction. As expected, the 1.6 and 1.1 kbp fragments were amplified from the wild-type and gene disruption mutant strains, respectively. However, both fragments were obtained from the complemented strain (Supporting Information Figure S1B). All transformants were further confirmed under LSCM.

### 3.2. BbAtg4 Contributes to Vegetative Growth

**Figure 2:**
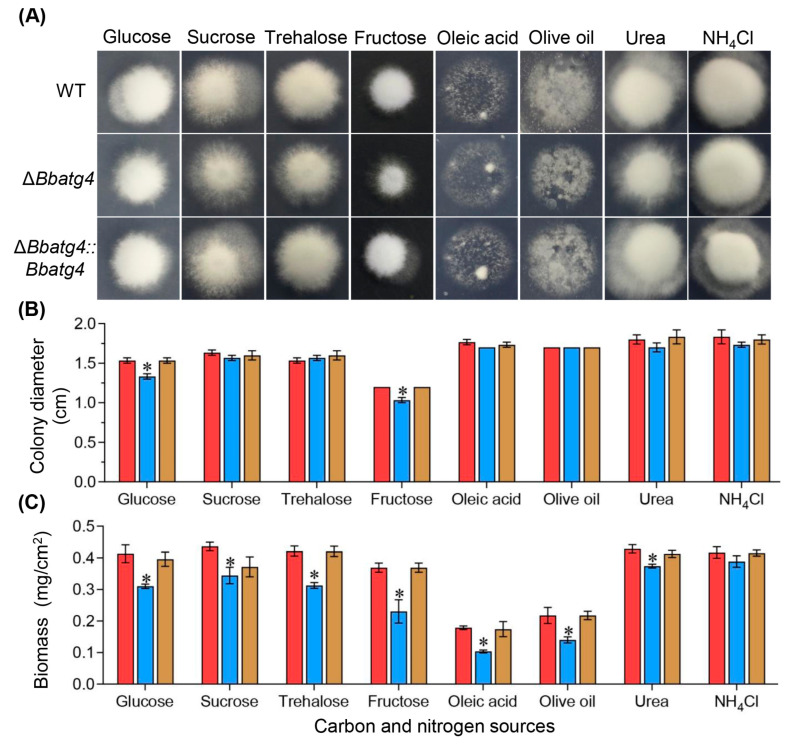
Effects of gene loss on fungal growth. Fungal strains were cultured on Sabouraud dextrose agar plates (SDAY) for conidiation. The conidia of indicated strain were inoculated on minimal medium supplemented with various carbon or nitrogen sources. After 7 d of incubation at 25 °C, colony morphologies were recorded (**A**), and diameters were examined (**B**). (**C**) To evaluate fungal biomass, conidial suspension was inoculated on SDAY plates and cultured for 7 d at 25 °C. The resultant mycelia were weighted after drying. Asterisks on the columns indicate a significant difference between gene disruption mutant and the wild-type or complemented strains (Student’s *t*-test; *, *p* <0.05). Error bars indicate the standard deviation from three replicates. Red bars: wild type; blue bars: Δ*Bbatg4*; brown bars: Δ*Bbatg4::Bbatg4*.

To determine the roles of BbAtg4 in nutrient utilization, vegetative growth was evaluated on different carbon or nitrogen sources (Figure 2A). After a 7-day incubation at 25 °C, Δ*Bbatg4* showed no significant reduction in colony diameter. Only on the culture medium using glucose and fructose as carbon sources, the colony diameter of Δ*Bbatg4* decreased slightly, with a reduction of 13.04% and 13.88%, respectively, when compared with that of the wild-type strain. However, the colony biomass of Δ*Bbatg4* mutant was significantly less than that for the wild-type and complementation mutant strains (Figure 2B). On various nutrients, the mycelial biomass of Δ*Bbatg4* decreased by 6.79 to 42.04%. These data indicated that BbAtg4 contributes to fungal vegetative growth (Figure 2C).

### 3.3. BbAtg4 Is Required for Conidial Germination under Nutrient-Limitation Condition

**Figure 3:**
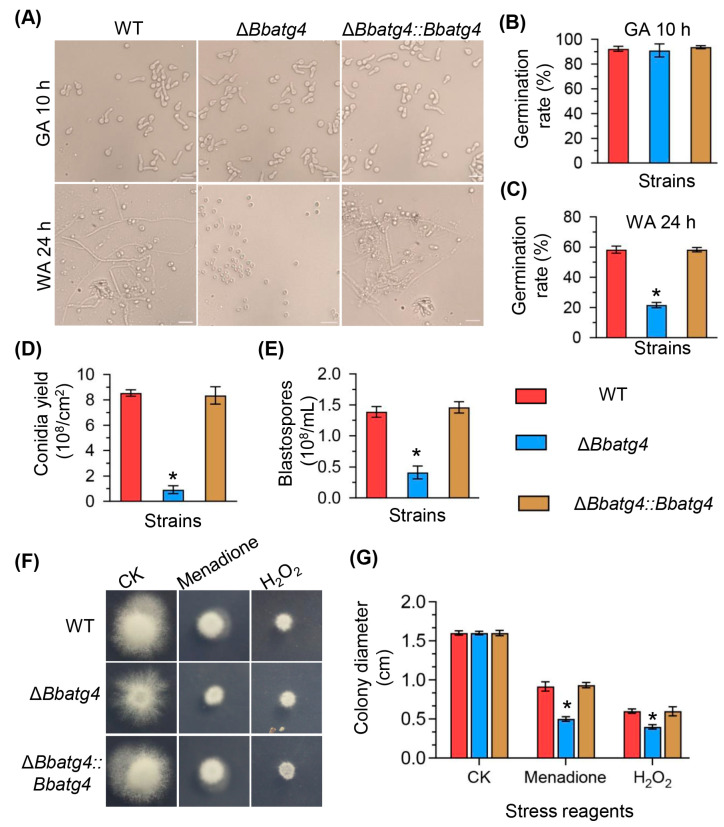
Effects of gene loss on conidial germination, stress response and development. Fungal conidia were harvested from Sabouraud dextrose agar plates (SDAY) after an incubation of 7 d. Conidia were inoculated on germination agar (GA) and water agar (WA) plates and cultured at 25 °C for 10 and 24 h, respectively. (**A**) Images for germination tubes were recorded at the sampling points. Germination level was quantified for conidia on GA (**B**) and WA (**C**). (**D**) Conidial production. Conidial suspension was inoculated on SDAY plates and cultured for 7 d at 25 °C. (**E**) Blastospore production. Conidial suspension was inoculated into SDB (SDAY without agar) and cultured for 3 d at 25 °C. To examine fungal response to oxidative stress, menadione and H_2_O_2_ were individually included in CzA plate, using plates without stressors as control (CK). Conidial suspension was inoculated on plate and cultured at 25 °C. Seven days later, colony morphologies were recorded (**F**), and their diameters were examined (**G**). Asterisks on the columns indicate a significant difference between gene disruption mutant and the wild-type or complemented strains (Student’s *t*-test; *, *p* < 0.05). Error bars indicate the standard deviation from three replicates.

The conidial germination was assayed on GM and WA plates, which represented nutrient and oligotrophic conditions, respectively (Figure 3A). On the GM plates (Figure 3B), after an incubation of 10 h, Δ*Bbatg4* did not exhibit a significant difference in the germination level when compared with the wild-type strain. On the WA plates (Figure 3C), at 24 h post-incubation, the germination levels for Δ*Bbatg4* were 21.67 ± 2.49%, which was significantly lower than that of the wild type (58.33 ± 3.30%) with a decrease of 79.03%. There was no significant difference between the wild-type and complementation strain.

### 3.4. BbAtg4 Is Involved in Fungal Development

At 7 days post-incubation under aerial conditions (Figure 3D), the Δ*Bbatg4* mutant exhibited a significant decrease in conidial yield on SDAY plates. The conidial yield of the mutant strain was 0.92 ± 0.44 × 10^8^ conidia/cm^2^ (mean ± SD), decreased by 89.20% when compared with the wild-type strain (8.54 ± 0.36 ×10^8^ conidia/cm^2^). There was no significant difference between the complementation strain and the wild type. Fungal development under submerged conditions was evaluated in the SDB medium. As shown in Figure 3E, blastospore concentration for Δ*Bbatg4* was 0.41 ± 0.15  × 10^8^ spores/mL (mean ± SD), with a decrease of 70.35% in comparison to that of the wild-type strain (1.39 ±  0.12  ×  10^8^ spores/mL). These results indicate that BbAtg4 plays an important role in the formation of conidia and blastospores on aerial surfaces and in liquid, respectively.

### 3.5. BbAtg4 Contributes to Fungal Resistance to Oxidative Stress

As shown in Figure 3F,G, without stress, after a 7-day incubation at 25 °C, Δ*Bbatg4* mutant showed no significant growth defects. On plates supplemented with menadione, the colony diameter for the wild-type and Δ*Bbatg4* mutant strains were 0.92 ± 0.08 and 0.50 ± 0.04 cm, respectively. Under H_2_O_2_ stress, the colony diameter for the wild-type and Δ*Bbatg4* mutant strains were 0.60 ± 0.04 and 0.40 ± 0.04 cm, respectively. These results indicated Δ*Bbatg4* displayed an enhanced sensitivity to oxidative stress.

### 3.6. BbAtg4 Is Important to Fungal Virulence

**Figure 4:**
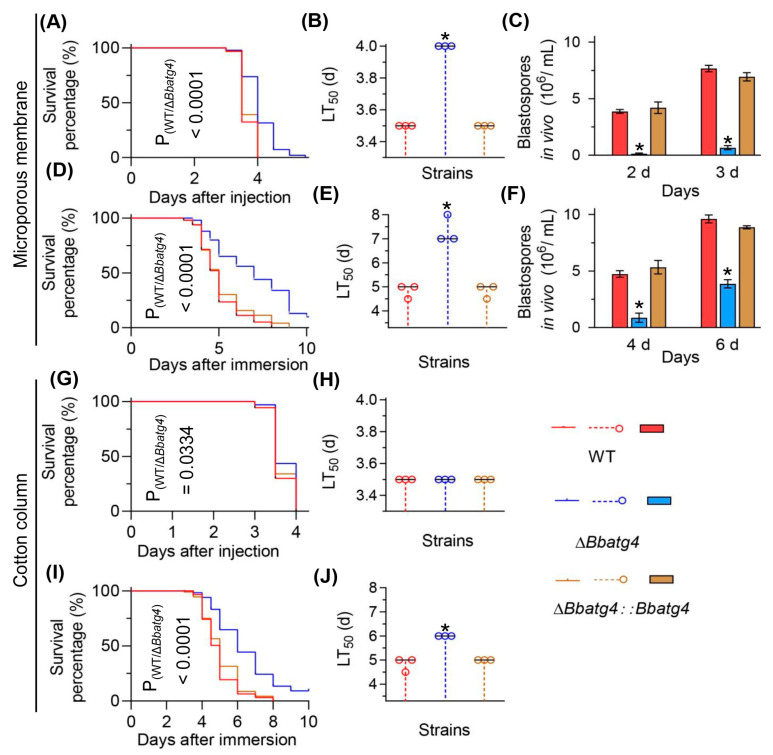
Insect bioassay. Fungal strains were cultured on SDAY plate for conidiation. To prepare conidial suspension, two filtration media were used, including cotton column and microporous membrane. Survival trends were noted for the insect hosts challenged with conidial suspensions via intrahemocoel injection (**A**,**G**) and topical application (**D**,**I**). Median lethal time (LT_50_) was calculated for bioassays with infection methods of intrahemocoel injection (**B**,**H**) and topical application (**E**,**J**). The in vivo blastospore production was examined in bioassays of intrahemocoel injection (**C**) and topical application (**F**).The fungal virulence indicated by LT_50_ value was significantly impaired by disruption of *BbATG4*. Asterisks on the columns indicate a significant difference between the Δ*Bbatg4* mutant and the wild-type or complemented strains (Student’s *t*-test; *, *p* <0.05). Error bars indicate the standard deviation from three replicates.

Two types of bioassay methods were used to evaluate fungal virulence against *G. mellonella* larvae. Firstly, we used the conidial suspension prepared with method 2 in bioassay (Figure 4A–F). In the intrahemocoel injection bioassay, the median lethal time (LT_50_) value of Δ*Bbatg4* was 4 days, delayed by 0.5 d when compared with that of the wild type (3.5 d) (Figure 4B). Notably, disruption of Δ*Bbatg4* led to a significant reduction in the yield of in vivo hyphal bodies (Figure 4C). The Δ*Bbatg4* mutant only produced 0.13 ± 0.09 × 10^6^ spores/mL at 2 days post-injection, with a reduction of 96.55% when compared with that of the wild-type strain (3.87 ± 0.25 × 10^6^ spores/mL). The in vivo blastospore yield of Δ*Bbatg4* strain increased at 3 d post-infection but still displayed a reduction of 91.30% when compared with that of the wild-type strain. In cuticle inoculation bioassay, the LT_50_ for the Δ*Bbatg4* mutant was 7.33  ±  0.47 d, significantly different from that of the wild-type strain (4.83  ±  0.24 d), with a delay of 51.72% (Figure 4E). After 4 d post-infection, the in vivo blastospore yield for Δ*Bbatg4* is 0.87 ±  0.57 × 10^6^ spores/mL and decreased by 81.69% when compared with that of the wild-type strain (4.73 ±  0.41 × 10^6^ spores/mL). The spore yield of the Δ*Bbatg4* strain (3.87 ±  0.52 × 10^6^ spores/mL) increased at 6 d post-infection but still displayed a reduction of 59.72% when compared with that of the wild-type strain (9.60 ±  0.49 × 10^6^ spores/mL) (Figure 4F).

Then, we used the conidial suspension prepared with method 1 in bioassay. As shown in Figure 4G–J, in the intrahemocoel injection bioassay, the LT_50_ value of Δ*Bbatg4* was 3.5 d and identical to that of the wild-type strain (3.5 d). In topical infection bioassay, the LT_50_ of Δ*Bbatg4* was only delayed by 1.27 d when compared to that of the wild-type strain. These results indicated that conidial suspension prepared with different methods displays different effects on the outcome of bioassay.

### 3.7. Autophagy Is Crucial for the Differentiation of Spore-Formation Structures

**Figure 5:**
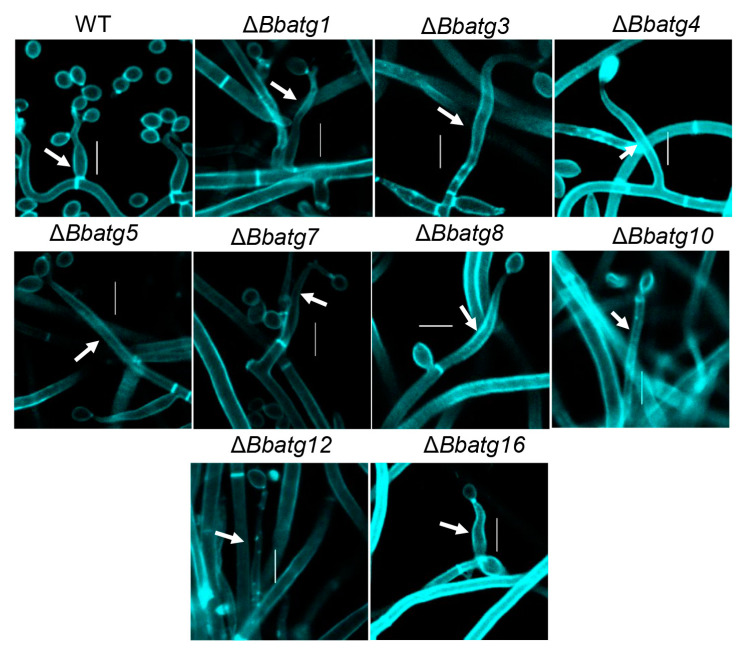
Microscopic view of conidium-producing structures. The wild type (WT) and its autophagy-null strains were cultured on SDAY plates at 25 °C. Five days later, mycelia/conidiophores were sampled and stained with calcofluor white. Images were taken under a fluorescent microscope. In all autophagy-null strains, conidiophores were significantly compromised, and very few conidia were observed. Bars: 5 µm.

Previous studies have shown that the ‘core’ autophagic genes (*BbAtg1*, *BbAtg3*, *BbAtg5*, *BbAtg7*, *BbAtg8*, *BbAtg12* and *BbAtg16*) are essential for spore formation in *B. bassiana* [4,9,24,25,26]. In this study, we examined the spore-producing structures in mutant deficient of the above *ATGs*. As shown in Figure 5, on the aerial surface, disruption of any one of the above *ATGs* resulted in significant impairment in fungal conidiophores. Microscopic examination indicated that the wild type produced abundant numbers of ‘bottle’ shaped conidiophores at 4 days post-incubation (dpi). However, at 7dpi, the autophagy-null strains produced the elongated and emaciated conidiophores, and very few conidia were observed.

**Figure 6:**
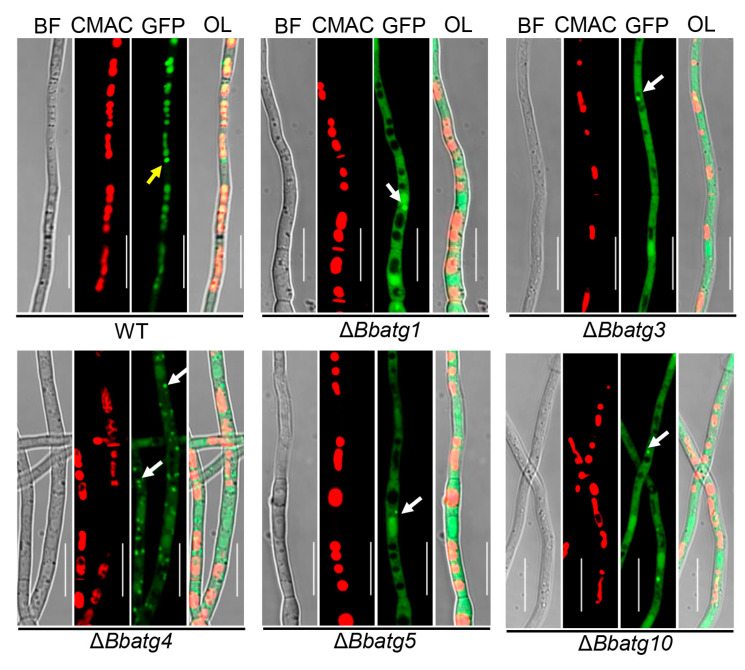
The autophagic process in aerial mycelia of *B. bassiana*. Fusion gene *GFP-ATG8* was transformed into the wild type (WT) and its autophagy-null strains. Conidial suspension of the indicated transformant was inoculated on SDAY plate and cultured for 3.5 d at 25 °C. The resultant mycelia were stained with CMAC (for vacuole), and autophagic process was examined under a fluorescent microscope. Autophagic signals were observed in the vacuoles of WT (yellow arrow), and Atg8 aggregates were only seen in cytosol of autophagy-null strains. BF: bright field; OL: overlapped. Scale bars: 10 μm.

During conidiation, autophagic flux was indicated with the fusion protein GFP-Atg8 [25,26,27]. Green signals were consistent with blue signals from the vacuole-specific dye (CMAC) in the wild-type strain. In Δ*Bbatg1*, Δ*Bbatg3*, Δ*Bbatg4*, Δ*Bbatg5* and Δ*Bbatg10* mutant strains, the GFP signals persisted in the cytoplasm. Notably, in gene disruption mutants, cytoplasmic BbAtg8 proteins aggregate into punctate aggregates, and more punctate signals were observed in Δ*Bbatg4* (Figure 6). Autophagy is also crucial for the differentiation of blastospores [27]. During the period of blastospore production, autophagic flow also exhibits the same trend as that involved in the period of conidia production (Figure S2).

### 3.8. Re-Examine the Virulence of Autophagy-Null Mutants

**Figure 7:**
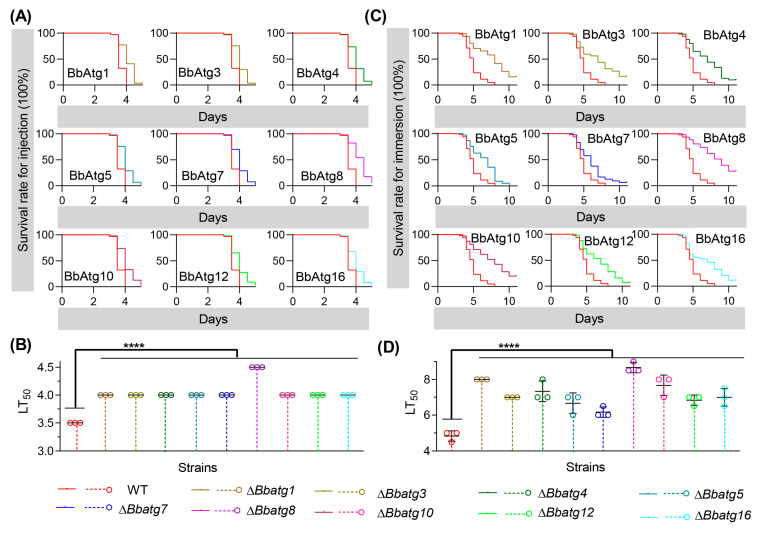
Re-examine conidial virulence. The wild type (WT) and its autophagy-null strains were cultured on SDAY plate for conidiation. To prepare conidial suspension, the mycelia were removed by filtering through microporous membrane. Survival trends were noted for the insect hosts challenged with conidial suspensions via intrahemocoel injection (**A**) and topical application (**C**). Median lethal time (LT_50_) was calculated for bioassays with infection methods of intrahemocoel injection (**B**) and topical application (**D**). Asterisks indicate a significant difference between the autophagy-null and the wild-type strains (Student’s *t*-test; ****, *p* < 0.0001). Error bars indicate the standard deviation from three replicates.

For autophagy-null strains, their conidia yield is very low, and it is hard to remove hyphal fragments from conidia. In this study, we introduced the method of membrane filtration in preparing conidial suspension and re-examined the virulence of autophagy-null mutants that have been published (Figure 7).

In the intrahemocoel injection bioassay (Figure 7A), all disruptants killed all bioassay insects, and all survival trends caused by autophagy-null mutants showed significant differences from that of the wild-type strain. In addition, there was a significant difference in the survival curve (Table 1) between the wild-type and individual mutant strains. As shown in Figure 7B, the median lethal time (LT_50_) value of the wild type was 3.5 d. The LT_50_ values of Δ*Bbatg1*, Δ*Bbatg3*, Δ*Bbatg4*, Δ*Bbatg5*, Δ*Bbatg7*, Δ*Bbatg10*, Δ*Bbatg12* and Δ*Bbatg16* were delayed to 4 d, while LT_50_ of Δ*BbAtg8* was delayed to 4.5 d. In topical application bioassay (Figure 7C), the wild type killed all tested hosts and exhibited a host survival curve that was significantly different from those caused by gene disruption mutants. As shown in Figure 7D, LT_50_ for the wild-type strain was 4.83  ±  0.24 d. Whereas, the LT_50_ values of Δ*Bbatg1*, Δ*Bbatg3*, Δ*Bbatg4*, Δ*Bbatg5*, Δ*Bbatg7*, Δ*Bbatg*8, Δ*Bbatg10*, Δ*Bbatg12* and Δ*Bbatg16* were 8.00, 7.00, 7.33, 6.67, 6.17, 8.87, 7.67, 6.83 and 7.00 d, respectively.

## 4. Discussion

In filamentous fungi, autophagy plays important roles in many physiological processes, including vegetative growth, development, lifespan and pathogenicity [21,28]. During the autophagic process, the ‘core’ ATG genes are indispensable for autophagy initiation and development, which are conserved in eukaryotes [29,30]. Autophagy has been linked to the whole lifecycle of *B. bassiana* [31]. In the present study, a homolog of yeast Atg4 was functionally analyzed in *B. bassiana*. The results demonstrated that BbAtg4 is required for the autophagic process and is involved in fungal growth, stress response, development and virulence. 

In *B. bassiana*, there exists a single Atg4 through sequence alignment analysis. Domain annotation uncovered that Atg4 contains a domain of Peptidase_C54 which is prevalent in Atg4 homologs from other fungal species. This finding reinforces that Atg4 is evolutionarily conserved in fungal species with different lifestyles [32]. As expected, BbAtg4 is required for autophagy in *B. bassiana*. Similarly, *A. oryzae* Atg4 is indispensable for autophagosome formation [12]. In *B. bassiana*, disruption of *BbATG4* significantly impairs conidial germination under starvation induction. This result is consistent with the *BbATG8* role in autophagy [31]. During autophagy, the cysteine protease Atg4 exposes the glycine residue at the extreme C-terminus of Atg8, and finally, Atg8 is activated through conjugation with PE [11]. The tripeptide at the C-terminus of BbAtg8 is essential for autophagy but not indispensable for its interaction with other proteins [21]. More BbAtg8 aggregates are present in the Δ*Bbatg4* strain than in other autophagy-null strains. This attributes to the Atg4 roles in the processing of Atg8 for maturation. Therefore, BbAtg4 acts as a functional ortholog of yeast Atg4 in *B. bassiana* autophagy.

In *B. bassiana*, autophagy is associated with the whole lifecycle of the fungus [21,31]. Conidial germination is a critical step for successful infection by *B. bassiana* [4]. Similar to other *ATG* genes, *BbATG4* is required for conidial germination under oligotrophic conditions but not for germination under nutrient-replete conditions. BbAtg4 contributes to fungal growth, and its loss impairs the accumulation of biomass but does not affect radial growth. As for Δ*Bbatg4*, the LT_50_ in the topical infection assay showed a significant delay when compared to that in the intrahemocoel injection bioassay. This suggests that BbAtg4 links autophagy to nutrient supply, which is critical for conidial germination on the host cuticle and follow-up invasive growth. Autophagy mediates the recycling of cellular nutrients during fungal growth and differentiation [30]. As a key regulator in autophagy, BbAtg7 contributes to fungal radial growth on chitin [25]. This result suggests that Atg proteins perform different roles in fungal growth with possible non-autophagic roles. 

In host hemocoel, the *B. bassiana* undergoes dimorphic transition and combat with different stresses caused by insect immune defense [33]. Like other *ATG* genes (e.g., *ATG1*, *ATG5* and *ATG8*) [4,31], BbAtg4 contributes to dimorphic change in *B. bassiana*. Additionally, BbAtg4 significantly contributes to the resistance of *B. bassiana* to oxidative stress, which is observed for other *ATG* genes (e.g., *ATG8* and *ATG11*) [31,33]. Thus, the reduced virulence of *B. bassiana* in intrahemocoel bioassay might be the combined defects of dimorphism and oxidation tolerance. In addition, BbAtg4 plays a more important role in fungal virulence through cuticle infection. This observation is also noted for other autophagy-related genes, which reinforces that autophagy is critical for the establishment of fungal infection on the host cuticle owing to autophagic roles in the mobilization of the endogenous reserve during conidial germination [31]. Two methods for conidial suspension preparation resulted in different results in bioassay. This might be due to the lower efficiency of the cotton column in removing the fragmented hyphae than the microporous membrane. In this study, we improved and standardized the methods used in preparing conidial suspension with a microporous membrane, which increase the comparability of bioassay data. In *B. cinerea*, Atg4 significantly contributes to virulence [13]. In *F. graminearum*, Atg4 contributes to fungal development, deoxynivalenol production and virulence [14]. In *M. oryzae*, Atg4 mediates appressorial maturation and pathogenesis [15]. In *M. robertsii*, Atg4 mediates lipid metabolism and virulence [16]. These results suggest that autophagy and its related genes mediate divergent mechanisms involved in fungal pathogenesis. 

A well-known role of *ATG*s in filamentous fungi is their involvement in conidiation [28]. All tested *ATG* genes have a convergent role in maintaining the conidiophore morphology, which is due to their autophagic roles. BbAtg4 contributes to approximately 90% of conidial capacity in *B. bassiana*, which is similar to that noted for BbAtg1 [31]. For aerial conidiation, BbAtg3, BbAtg5, BbAtg7 and BbAtg11 have similar roles. Their disruption mutants display a reduction of approximately 70% in conidial yield [4,9,24,25,33]. BbAtg12 and BbAtg16 have more important roles in conidiation, and their losses result in approximately 80% reduction in conidial yield [26]. As for *B. bassiana*, the conidiation process is critical for fungal survival and subsequent infection cycles [21]. From this perspective, Atg4, together with other *ATG*s, is significantly involved in maintaining the infection cycle of *B. bassiana*. Increasing evidence suggests that *ATG* genes perform a variety of non-autophagic roles [34]. In addition, Bbatg5 contributes to maintaining conidial size [4]. Thus, these findings reinforce that *B. bassiana ATG* genes might mediate the divergent pathways in fungal differentiation beyond their common roles in autophagy.

## 5. Conclusions

In summary, BbAtg4 is indispensable for the fungal autophagic process during development. This gene contributes to stress response (starvation and oxidation), spore production and virulence in *B. bassiana*. This study provides more understanding of the effects of autophagy on physiology in filamentous fungi.

## Figures and Tables

**Table 1:** Log-rank tests were performed between the wild-type and individual mutant strains.

Strains	Injection Infection		Topical Infection	
	*χ^2^* Value	*p* Value	*χ^2^* Value	*p* Value
Δ*Bbatg1*	57.38	<0.0001	61.33	<0.0001
Δ*Bbatg3*	49.93	<0.0001	29.54	<0.0001
Δ*Bbatg4*	44.54	<0.0001	44.02	<0.0001
Δ*Bbatg5*	49.62	<0.0001	29.24	<0.0001
Δ*Bbatg7*	40.10	<0.0001	18.75	<0.0001
Δ*Bbatg8*	74.79	<0.0001	99.63	<0.0001
Δ*Bbatg10*	47.54	<0.0001	60.45	<0.0001
Δ*Bbatg12*	31.18	<0.0001	36.15	<0.0001
Δ*Bbatg16*	37.12	<0.0001	23.89	<0.0001

## Data Availability

Not applicable.

## Supplementary Materials

The following supporting information can be downloaded at: https://www.mdpi.com/article/10.3390/jof9050543/s1, Figure S1: Gene disruption and complementation in *B. bassiana*; Figure S2: The autophagic process in submerged mycelia of *B. bassiana*; Table S1: Primers used in this study.

## References

[1] De la Cruz Q.R., Roussos S., Hernandez D., Rodriguez R., Castillo F., Aguilar C.N. (2015). Challenges and opportunities of the biopesticides production by solid-state fermentation: Filamentous fungi as a model. Crit. Rev. Biotechnol..

[2] Maistrou S., Natsopoulou M.E., Jensen A.B., Meyling N.V. (2020). Virulence traits within a community of the fungal entomopathogen *Beauveria*: Associations with abundance and distribution. Fungal Ecol..

[3] Ortiz-Urquiza A., Keyhani N.O. (2013). Action on the surface: Entomopathogenic fungi versus the insect cuticle. Insects.

[4] Zhang L., Wang J., Xie X.Q., Keyhani N.O., Feng M.G., Ying S.H. (2013). The autophagy gene *BbATG5*, involved in the formation of the autophagosome, contributes to cell differentiation and growth but is dispensable for pathogenesis in the entomopathogenic fungus *Beauveria bassiana*. Microbiology.

[5] Wanchoo A., Lewis M.W., Keyhani N.O. (2009). Lectin mapping reveals stage-specific display of surface carbohydrates in in vitro and haemolymph-derived cells of the entomopathogenic fungus *Beauveria bassiana*. Microbiology.

[6] Ding J.L., Hou J., Feng M.G., Ying S.H. (2020). Transcriptomic analyses reveal comprehensive responses of insect hemocytes to mycopathogen *Beauveria bassiana*, and fungal virulence-related cell wall protein assists pathogen to evade host cellular defense. Virulence.

[7] Meyling N.V., Pell J.K., Eilenberg J. (2006). Dispersal of *Beauveria bassiana* by the activity of nettle insects. J. Invertebr. Pathol..

[8] Farré J.C., Subramani S. (2016). Mechanistic insights into selective autophagy pathways: Lessons from yeast. Nat. Rev. Mol. Cell Biol..

[9] Ying S.H., Liu J., Chu X.L., Xie X.Q., Feng M.G. (2016). The autophagy-related genes *BbATG1* and *BbATG8* have different functions in differentiation, stress resistance and virulence of mycopathogen *Beauveria bassiana*. Sci. Rep..

[10] Meijer W.H., van der Klei I.J., Veenhuis M., Kiel J.A.K.W. (2007). ATG genes involved in non-selective autophagy are conserved from yeast to man, but the selective Cvt and pexophagy pathways also require organism-specific genes. Autophagy.

[11] Nakatogawa H. (2020). Mechanisms governing autophagosome biogenesis. Nat. Rev. Mol. Cell Biol..

[12] Kikuma T., Kitamoto K. (2011). Analysis of autophagy in *Aspergillus oryzae* by disruption of *Aoatg13*, *Aoatg4*, and *Aoatg15* genes. FEMS Microbiol. Lett..

[13] Liu N., Ren W., Li F., Chen C., Ma Z. (2019). Involvement of the cysteine protease BcAtg4 in development and virulence of *Botrytis cinerea*. Curr. Genet..

[14] Lv W., Wang C., Yang N., Que Y., Talbot N.J., Wang Z. (2017). Genome-wide functional analysis reveals that autophagy is necessary for growth, sporulation, deoxynivalenol production and virulence in *Fusarium graminearum*. Sci. Rep..

[15] Kershaw M.J., Talbot N.J. (2009). Genome-wide functional analysis reveals that infection-associated fungal autophagy is necessary for rice blast disease. Proc. Natl. Acad. Sci. USA.

[16] Duan Z., Chen Y., Huang W., Shang Y., Chen P., Wang C. (2013). Linkage of autophagy to fungal development, lipid storage and virulence in *Metarhizium robertsii*. Autophagy.

[17] Ding J.L., Lin H.Y., Feng M.G., Ying S.H. (2020). Mbp1, a component of the *MluI* cell cycle box-binding complex, contributes to morphological transition and virulence in the filamentous entomopathogenic fungus *Beauveria bassiana*. Environ. Microbiol..

[18] Wang J.J., Peng Y.J., Ding J.L., Feng M.G., Ying S.H. (2020). Mitochondrial fission is necessary for mitophagy, development and virulence of the insect pathogenic fungus *Beauveria bassiana*. J. Appl. Microbiol..

[19] Guo N., Qian Y., Zhang Q., Chen X., Zeng G., Zhang X., Mi W., Xu C., St Leger R.J., Fang W. (2017). Alternative transcription startsite selection in Mr-OPY2 controls lifestyle transitions in the fungus *Metarhizium robertsii*. Nat. Commun..

[20] Ding J.L., Li X.H., Lei J.H., Feng M.G., Ying S.H. (2022). Succinate dehydrogenase subunit C contributes to mycelial growth and development, stress response, and virulence in the insect parasitic fungus *Beauveria bassiana*. Microbiol. Spectr..

[21] Ding J.L., Lin H.Y., Hou J., Feng M.G., Ying S.H. (2023). The entomopathogenic fungus *Beauveria bassiana* employs autophagy as a persistence and recovery mechanism during conidial dormancy. mBio.

[22] Peng Y.J., Hou J., Zhang H., Lei J.H., Lin H.Y., Ding J.L., Feng M.G., Ying S.H. (2022). Systematic contributions of CFEM domain-containing proteins to iron acquisition are essential for interspecies interaction of the filamentous pathogenic fungus *Beauveria bassiana*. Environ. Microbiol..

[23] Wang F., Sethiya P., Hu X., Guo S., Chen Y., Li A., Tan K., Wong K.H. (2021). Transcription in fungal conidia before dormancy produces phenotypically variable conidia that maximize survival in different environments. Nat. Microbiol..

[24] Lin H.Y., Ding J.L., Peng Y.J., Feng M.G., Ying S.H. (2022). Proteomic and phosphoryproteomic investigations reveal that autophagy-related protein 1, a protein kinase for autophagy initiation, synchronously deploys phosphoregulation on the ubiquitin-like conjugation system in the mycopathogen *Beauveria bassiana*. mSystems.

[25] Lin H.Y., Wang J.J., Feng M.G., Ying S.H. (2019). Autophagy-related gene *ATG7* participates in the asexual development, stress response and virulence of filamentous insect pathogenic fungus *Beauveria bassiana*. Curr. Genet..

[26] Hou J., Wang J.J., Lin H.Y., Feng M.G., Ying S.H. (2020). Roles of autophagy-related genes in conidiogenesis and blastospore formation, virulence, and stress response of *Beauveria bassiana*. Fungal Biol..

[27] Ding J.L., Zhang H., Feng M.G., Ying S.H. (2023). Divergent physiological functions of four Atg22-like proteins in conidial germination, development, and virulence of the entomopathogenic fungus *Beauveria bassiana*. J. Fungi.

[28] Deng Y.Z., Qu Z., Naqvi N.I. (2012). Role of macroautophagy in nutrient homeostasis during fungal development and pathogenesis. Cells.

[29] Mizushima N. (2020). The ATG conjugation systems in autophagy. Curr. Opin. Cell Biol..

[30] Pollack J.K., Harris S.D., Marten M.R. (2009). Autophagy in filamentous fungi. Fungal Genet. Biol..

[31] Ying S.H., Feng M.G. (2019). Insight into vital role of autophagy in sustaining biological control potential of fungal pathogens against pest insects and nematodes. Virulence.

[32] Meijer A.J., Codogno P. (2007). AMP-activated protein kinase and autophagy. Autophagy.

[33] Ding J.L., Peng Y.J., Chu X.L., Feng M.G., Ying S.H. (2018). Autophagy-related gene *BbATG11* is indispensable for pexophagy and mitophagy, and contributes to stress response, conidiation and virulence in the insect mycopathogen *Beauveria bassiana*. Environ. Microbiol..

[34] Cadwell K., Debnath J. (2017). Beyond self-eating: The control of non-autophagic functions and signaling pathways by autophagy-related proteins. J. Cell Biol..

