# Supplementary material for: Autophagy-Related Gene 4 Participates in the Asexual Development, Stress Response and Virulence of Filamentous Insect Pathogenic Fungus Beauveria bassiana
Source: J Fungi (Basel). 2023 May 6;9(5):543. doi: 10.3390/jof9050543 (PMC10219160; doi:10.3390/jof9050543)

Figure S1. Gene disruption and complementation in *B. bassiana*. (A) Diagram for plasmid construction. The upstream and downstream flanking sequences of target gene are amplified by primer pair P1/P2 and P3/P4, respectively. The resultant fragments are fused to cassette of *bar* gene, and then is then connected with *GFP* cassette in disruption vector. To complement the gene loss, the full length of *BbATG4* was amplified with primer pair P5/P6 and cloned into a plasmid with *Nat* cassette. All fungal transformants were screened by PCR with the primer pair P7/P8. GFP: green fluorescent protein. (B) PCR reaction to screen the gene disruption and complemented mutants. Lane1: wild type, lane 2: disruption mutant, lane 3: complemented strain, lane M: DNA marker.

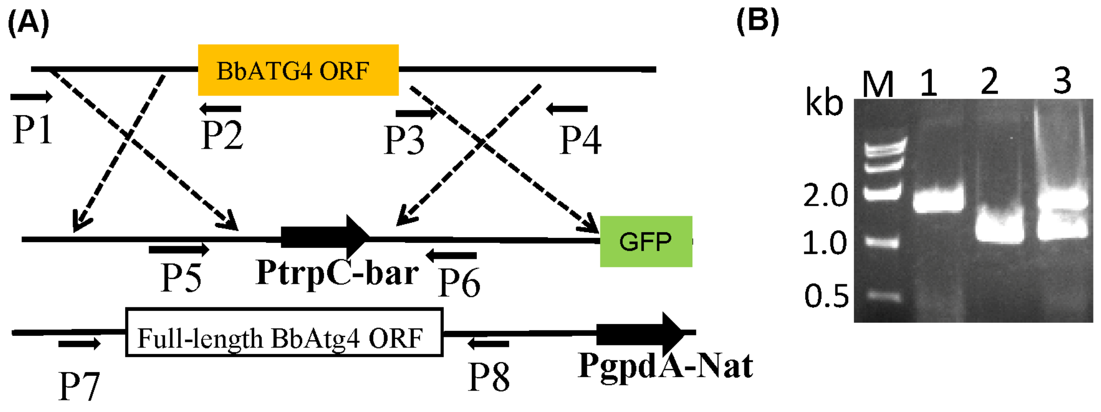

Supplement: Supplementary file 1 [file jof-09-00543-s001.zip › Figure S1.pdf]
