# Supplementary material for: Autophagy-Related Gene 4 Participates in the Asexual Development, Stress Response and Virulence of Filamentous Insect Pathogenic Fungus Beauveria bassiana
Source: J Fungi (Basel). 2023 May 6;9(5):543. doi: 10.3390/jof9050543 (PMC10219160; doi:10.3390/jof9050543)

Figure S2. The autophagic process in submerged mycelia of *B. bassiana*. Fusion gene *GFP-ATG8* was transformed into the wild-type (WT) and its autophagy-null strains. Conidial suspension of the indicated transformant was inoculated into SDB media and cultured for 2 d at 25°C. The resultant mycelia were stained with vacuole-specific CMAC and autophagic process was examined under a fluorescent microscope. Autophagic signals were observed in the vacuoles of WT (yellow arrow), and Atg8 aggregates were only seen in the cytosol of autophagy-null strains. BF: bright field; OL: overlapped. Scale bars: 10 μm.

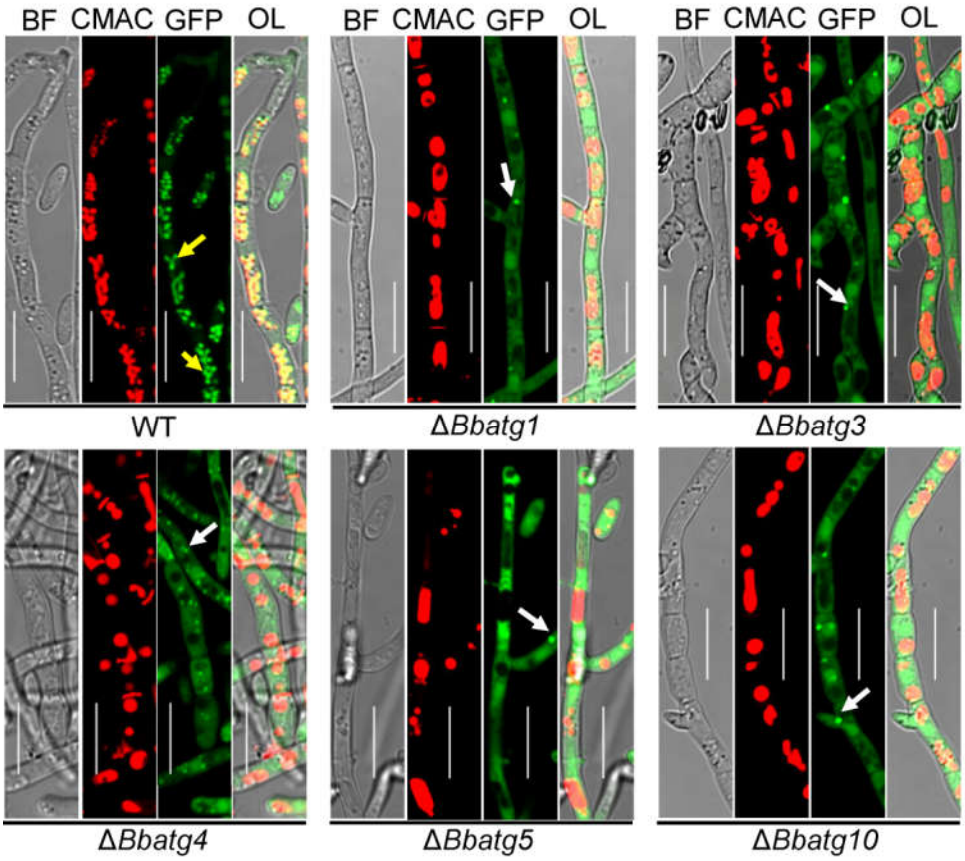

Supplement: Supplementary file 1 [file jof-09-00543-s001.zip › Figure S2.pdf]
