# Supplementary material for: Autophagy-Related Gene 4 Participates in the Asexual Development, Stress Response and Virulence of Filamentous Insect Pathogenic Fungus Beauveria bassiana
Source: J Fungi (Basel). 2023 May 6;9(5):543. doi: 10.3390/jof9050543 (PMC10219160; doi:10.3390/jof9050543)
Supplement: Supplementary file 1 [file jof-09-00543-s001.zip › Table S1.pdf]

Table S1. **Primers used in this study.**

Primers	Paired sequences (5′–3′)*	Purpose
P1	CGAGCTGTACAAGTAACCCGGGCCAACATTCCATCGTCCTC	Amplifying 5′-fragment of *BbATG4*
P2	TTGGCTGCAGGTCGACGGATCCCTGCTCGTTGTCTGTAGTGC	
P3	CGACCCATGGCTCGAGTCTAGAGTGATGATGATATGGAGGATT	Amplifying 3′-fragment of *BbATG4*
P4	GGTGGTGGTGGCTAGCGTTAACTGAGTCGGAGGTGGAAAT	
P5	ACAGTACACGAGGACTTCTAGACCAACATTCCATCGTCCTC	Amplifying *BbATG4* for complementation
P6	GCCCTGCCCCTGAGAGGAATTCTGAGTCGGAGGTGGAAAT	
P7	CTCTGGAGCAAACGGGTAA	PCR screening
P8	ACATTTCTGCGGTCTACTCTGC	

*: The underlined sequence is required for recombination in preparing plasmid.
